# Burnout Syndrome Among Hospital Healthcare Workers During the COVID-19 Pandemic and Civil War: A Cross-Sectional Study

**DOI:** 10.3389/fpsyt.2020.579563

**Published:** 2020-12-11

**Authors:** Muhammed Elhadi, Ahmed Msherghi, Moutaz Elgzairi, Ayiman Alhashimi, Ahmad Bouhuwaish, Marwa Biala, Seraj Abuelmeda, Samer Khel, Ala Khaled, Ahmed Alsoufi, Amna Elmabrouk, Fatimah Bin Alshiteewi, Tasneem Ben Hamed, Bushray Alhadi, Sara Alhaddad, Ahmed Elhadi, Ahmed Zaid

**Affiliations:** ^1^Faculty of Medicine, University of Tripoli, Tripoli, Libya; ^2^Faculty of Medicine, University of Benghazi, Benghazi, Libya; ^3^Faculty of Medicine, Al-Jabal Al Gharbi University, Gherian, Libya; ^4^Faculty of Medicine, Tobruk University, Tobruk, Libya

**Keywords:** coronavirus disease, medical staff, professional burnout, COVID-19, psychiatric illness, pandemic, war exposure

## Abstract

**Objective:** We aimed to determine the prevalence of burnout among hospital healthcare workers in Libya during the coronavirus disease (COVID-19) pandemic and civil war.

**Methods:** A cross-sectional study was conducted from April 18 to May 2, 2020 among Libyan healthcare workers. Data on participant characteristics were collected with a specifically designed questionnaire. Burnout was assessed with the abbreviated Maslach Burnout Inventory (aMBI) comprising three subscales: emotional exhaustion (EE), depersonalization (DP), and personal accomplishment (PA), with each sub-scale score range from 0 to 18. For EE and DP, scores of 10 to 18 were regarded as “moderate to severe burnout.” PA was scored inversely, where a score ≤ 10 indicated severe burnout.

**Results:** The study yielded a sample size of 532 participants. Of these, 357 (67.1%) reported emotional exhaustion (EE Score ≥ 10), 252 (47.4%) reported depersonalization (DP score ≥ 10), and 121 (22.7%) reported a lower sense of personal accomplishment (PA score ≤ 10). Verbal abuse was experienced by 304 participants (57.1%) and physical abuse in 93 (17.5). Gender was associated with high emotional exhaustion and high depersonalization. Being 35 years or older was associated with high depersonalization. Professional specialty was significantly associated with high emotional exhaustion and depersonalization. Fear of COVID-19 infection was associated with high emotional exhaustion and high depersonalization.

**Conclusion:** The rising prevalence of mental disorders and inadequate availability of health services facilities during the COVID-19 pandemic and civil war demonstrated the need for healthcare policies to address the well-being of healthcare workers to decrease the risk of loss, suicide, and medical negligence.

## Introduction

In December 2019, a novel coronavirus designated as severe acute respiratory syndrome coronavirus 2 (SARS-CoV-2) was identified as the cause of severe viral pneumonia in Wuhan, a city in Hubei Province, China (1). This virus was recognized as a global pandemic on February 11, 2020 (2). By August 14, the World Health Organization recorded over twenty million cases of coronavirus disease (COVID-19), including more than 756,000 deaths (3, 4).

Since the emergency of the first case of COVID-19 was detected in Libya on March 24, followed by a substantial increase in the number of cases. By August 14, more than 6,611 cases were confirmed, with the death toll exceeding 132 (5).

Research indicates that frontline healthcare staff involved in the management and diagnosis of COVID-19 are at risk of experiencing psychiatric disturbances and deteriorating mental health (6). This may be a result of various challenges, such as shortages of personal protective equipment, scarcity of appropriate medications, risk of infecting family members, expectations of inadequate assistance, and fear of contracting the virus. Along with the financial difficulties that physicians are facing in many countries, these factors place healthcare workers under considerable pressure, threatening their mental well-being (7, 8).

Several studies have identified a correlation between mental health issues and the COVID-19 pandemic in healthcare workers. A recent study conducted in China from January 29, 2020, to February 3, 2020, to assessed the mental health status of physicians and nurses, and found that they demonstrated a high prevalence of anxiety, depression, and insomnia (6). Further, a study conducted in Singapore from February 19, 2020, to March 13, 2020, observed increased psychological distress, anxiety, and depression among healthcare workers during COVID-19 (9).

However, there is little data available on physician burnout during the pandemic. Burnout is defined as a medical condition of physical and mental fatigue associated with work or care-providing activities (10, 11). Burnout involves cognitive fatigue, depersonalization, and a diminished sense of success (12). Since physicians endure an extremely taxing working environment, burnout syndrome among healthcare workers has attracted major interest in recent years.

Research supports that physicians are at higher risk of burnout due to exposure to emotional pressure beyond the level experienced in most other professions (13). Furthermore, burnout has been linked to decreased efficiency and diminished work satisfaction among physicians (14). Subsequently, irritability and dissatisfaction may impact individual's sense of well-being and willingness to function fully at work, negatively affecting the ability of physicians to care for patients (15). In addition, physician burnout has been linked to increased risk of medical errors, which also has a harmful effect on patient outcomes (16). Worryingly, burnout has been associated with suicidal risk and elevated levels of depression (17–20). The condition has also been linked to physiological issues, such as increased risk of cardiovascular diseases (21, 22), and inflammation biomarker elevation (23).

A further exacerbating factor for Libyan physicians is that since 2011, Libya has suffered from several civil wars and financial crises that can potentially lead to conflict-related traumatic events and higher rates of mental disorders (24, 25). In addition, due to the absence of a formal psychiatric training program, Libya lacks adequate mental healthcare facilities; currently having 0.2 psychiatrists and 0.05 psychiatric nurses per 100,000 people (26, 27).

Therefore, we believe that healthcare workers in Libya are at greater risk for burnout syndrome and lower quality of life during the COVID-19 pandemic. We aimed to determine the prevalence of, and factors associated with, burnout syndrome among Libyan healthcare workers during the COVID-19 pandemic in a time of civil war.

## Methods

This was a cross-sectional study.

### Participants

Healthcare professionals working in Libyan hospitals were recruited for the study from April 18, 2020, to May 2, 2020. Data were collected via a questionnaire that was distributed among 20 major hospitals in printed and electronic format, via mobile messages and emails. Inclusion criteria were as follows: participants must have worked in late March and April, and must have worked in either surgery, internal medicine, intensive care, or emergency departments. Exclusion criteria were as follows: having patients with mental illnesses or severe chronic diseases such as advanced diabetes, hypertension, and tuberculosis. Participants with missing data, incomplete Abbreviated Maslach Burnout Inventory (aMBI), or those with a history of mental illness were excluded from the analysis.

### Measures

The questionnaire contained two sections. The first section was developed specifically for the study and comprised participant demographic characteristics, marital status, years of work experience, work shifts, number of working hours per week, illicit drug use and smoking history, employment status, educational level, perspectives on COVID-19, social stigmatization, the effects of the civil war, internal displacement, transportation-related issues, physical and verbal abuse of physicians.

The second section contained the English version of the Abbreviated Maslach Burnout Inventory (aMBI), which is a nine-item scale developed for and most commonly used in the detection of burnout among physicians (28–31). The aMBI comprises three subscales: emotional exhaustion (EE, emotional depletion due to job demand and continuous work-related stress), depersonalization (DP, impersonal response toward the recipient service), and personal accomplishment (PA, the degree of personal competence, achievement, and job satisfaction). Each subscale contains three items. Responses are based on a seven-point Likert scale, ranging from 0 (“never”) to 6 (“every day”). For EE and DP, a higher score indicates greater burnout, and for PA, a higher score indicates a greater sense of accomplishment. Therefore, high EE and DP scores, and a low PA score indicated a higher level of burnout. Overall burnout was taken as the sum of EE and DP scores.

The scores of each subscale ranged from 0 to 18. For EE and DP, scores of 0 to 9 were categorized as “no to low burnout” and scores of 10 to 18 were regarded as “moderate to severe burnout.” This was the inverse for PA because higher PA scores indicate less burnout. For PA, a score ≤ 10 indicates severe burnout. The score for each item was summed for each physician.

The aMBI is a reliable tool to measure burnout among physicians and has been validated in several previous studies (32–37). The Cronbach's alpha coefficient scores for each subscale were as follows: emotional exhaustion α = 0.89, depersonalization α = 0.76, personal accomplishment α = 0.72, and overall burnout α = 0.81.

### Statistical Analysis

Data did not follow normal distribution according to Shapiro-Wilk test. Confirmatory factor analysis of the Abbreviated Maslach Burnout Inventory (aMBI) was assessed using structural equation modeling (SEM) as previously published (38), and the models were tested using IBM® SPSS® Amos™ 24 (IBM Corp., Armonk, NY, USA). This yielded several measures as follows: χ2 minimum fit test as in inferential testing of the model. The root means square error of approximation (RMSEA) determine the lack of fit due to reliability (39), where it provides fit per degree of freedom of the model with 0.05 or less desirable as indicating good fit model. Adjusted goodness of it index (AGFI) and the goodness of fit index (GFI) adjust for the number of estimated with a range from 0 to 1 with 0.9 or more as a desirable indicator of good fitting model. Also, comparative fit index (CFI) will be used to assess fit related to null modeling using noncentrality parameters (40). CFI range from 0 to 1 with 0.9 or more as an indicator of good fitting model. The standardized root mean square residual (RMR) was used as the average of the differences between sample correlations and estimated population correlations, with a value range from 0 to 1; where <0.08 indicative of fitting model (41).

Differential item functioning (DIF) using multiple indicator multiple cause (MIMIC) model was performed on latent factors (EE, DP, PA) of the best fitting model on the “online vs. paper” variable to see if there were statistically significant coefficients between the two methods of data collection which may necessitate splitting of the samples or whether both ways yield similar valid results.

Baseline characteristics and working conditions for men and women were compared using the Mann-Whitney U test for continuous variables and the chi-square test for categorical variables. Fisher's exact test was used to compare the burnout subscales with the demographic data. Phi (ϕ) was used to measure the strength of association of two dichotomous variables, while Cramer's V was used to measure the strength or association of more than two nominal variables. The aMBI level of internal consistency was determined by a Cronbach's alpha among study participants. A Spearman's rank-order correlation test was conducted to assess the relationship between emotional exhaustion, depersonalization, and personal accomplishment scores and study variables. Data entry and statistical analysis was performed using SPSS version 25.0 (IBM Corp., Armonk, NY, USA).

### Ethical Considerations

The study was approved by the Bioethics Committee at the Biotechnology Research Center in Libya. All participants provided consent before participating in the study.

## Results

A total of 532 out of 600 (88.66%) participants completed the questionnaires [353 (66.4%) online, 179 (33.6%) papers]. The mean age was 33.08 (SD = 7.25). The sample comprised 294 (55.3%) males and 238 (44.7%) females. Participants with incomplete questionnaire data were excluded from the analysis. Only responses from internal medicine (223; 41.9%), intensive care (64; 12%), emergency medicine (111; 20.9%), and surgical departments (134; 25.2%), and their subspecialties were included. The participants' baseline characteristics are presented in Table 1. A chi-square test for association was conducted between gender and subjects' basic characteristics. There was a statistically significant association between gender and marital status, living conditions, employment status, years of work experience, department, smoking, and physical abuse (*p* < 0.05). We used the validated English version of aMBI, and the scale was tested for internal consistency, as determined by a Cronbach's alpha of 0.76 for emotional exhaustion, α = 0.66 for depersonalization, α = 0.71 for personal accomplishment, and overall burnout α = 0.801.

**Table 1:** Baseline characteristics of study participants (*n* = 532).

**Variables**	**Total (%)**	**Women(%)**	**Men (%)**	**Phi (ϕ)/Cramer's V**	***P*-value**
	***n* = 532**	***n* = 238**	***n* = 294**		
Age range				0.07	0.087
<35	392 (73.7)	184 (77.3)	208 (70.7)		
≥35	140 (26.3)	54 (22.7)	86 (29.3)		
Marital status				0.09	0.038^*^
Married	223 (41.9)	88 (37)	135 (45.9)		
Not married (single, divorced, widow)	309 (58.1)	150 (63)	159 (54.1)		
Living arrangements				0.09	0.038^*^
With family	348 (65.4)	167 (70.2)	181 (61.6)		
Alone	184 (34.6)	71 (29.8)	113 (38.4)		
Employment status				0.28	<0.001^**^
Governmental sector	244 (45.9)	143 (60.1)	101 (34.4)		
Private sector	65 (12.2)	30 (12.6)	35 (11.9)		
Both	223 (41.9)	65 (27.3)	158 (53.7)		
Years of experience				0.14	0.019^*^
<3 years	231 (43.4)	114 (47.9)	117 (39.8)		
3–5 years	111 (20.9)	53 (22.3)	58 (19.7)		
5–15	143 (26.9)	48 (20.2)	95 (32.3)		
>15 years	47 (8.8)	23 (9.7)	24 (8.2)		
Department				0.34	<0.001^**^
Internal Medicine Departments	223 (41.9)	134 (56.3)	89 (30.3)		
Surgical Departments	134 (25.2)	25 (10.5)	109 (37.1)		
Emergency Medicine	111 (20.9)	54 (22.7)	57 (19.4)		
Intensive Care Units	64 (12)	25 (10.5)	39 (13.3)		
Smoking				0.4	<0.001^**^
Yes	96 (18)	2 (0.8)	94 (32)		
No	436 (82)	236 (99.2)	200 (68)		
Illicit drugs use				0.04	0.322
Yes	18 (3.4)	6 (2.5)	12 (4.1)		
No	514 (96.6)	232 (97.5)	282 (95.9)		
Stigmatization due to COVID-19				0.08	0.052
Yes	169 (31.8)	86 (36.1)	83 (28.2)		
No	363 (68.2)	152 (63.9)	211 (71.8)		
Internal displacement				0.003	0.941
Yes	173 (32.5)	77 (32.4)	96 (32.7)		
No	359 (67.5)	161 (67.6)	198 (67.3)		
Living in conflict area				0.05	0.246
Yes	176 (33.1)	85 (35.7)	91 (31)		
No	356 (66.9)	153 (64.3)	203 (69)		
Verbal abuse				0.02	0.725
Yes	304 (57.1)	134 (56.3)	170 (57.8)		
No	228 (42.9)	104 (43.7)	124 (42.2)		
Physical abuse				0.13	0.004^*^
Yes	93 (17.5)	29 (12.2)	64 (21.8)		
No	439 (82.5)	209 (87.8)	230 (78.2)		
Working hours per week, (mean ± SD)	53.26 ± 11.19	49.98 ± 8.93	55.91 ± 12.1		<0.001^**^
Number of shifts per month, mean ± SD	3.66 ± 0.71	3.52 ± 0.73	3.78 ± 0.67		<0.001^**^

SD, standard deviation.

* Significant at (p < 0.05);

** *Significant at (p < 0.001)*.

### Confirmatory Factor Analysis

**Figure 1:**
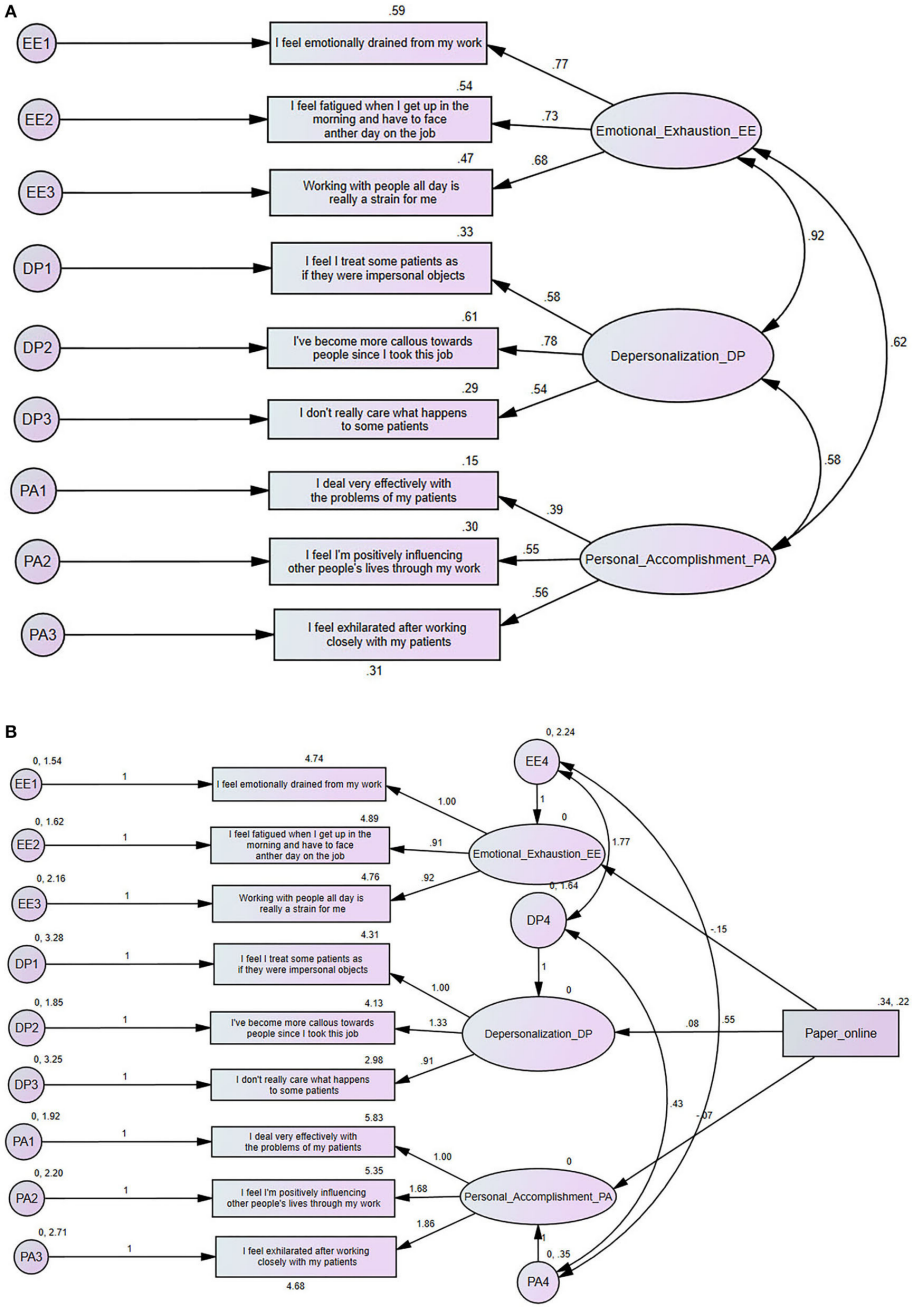
**(A)** Standardized parameter estimates for the factor structure of the Abbreviated Maslach Burnout Inventory (aMBI). **(B)** Multiple indicator multiple cause (MIMIC) structural equation model for for differential item functioning (DIF) based on paper or online method.

CFA was performed on aMBI items, and parameters were calculated by using maximum likelihood. Minimum was achieved, χ2 = 170.35, degree of freedom (df) = 24, *p* ≤ 0.001. While (GFI = 0.93, AGFI = 0.87, CFI = 0.88, RMSEA = 0.11, and RMR = 0.22). The results are summarized in Table 2 and Figure 1A provides an overview of the model. Testing measurement invariance was performed between paper and online collection method to see if there is any difference. Overall model, we found χ2 = 190.82, df = 48, *p* < 0.001. We found that CMIN/DF was found 3.97, GFI = 0.927, AGFI = 0.86, RMESEA = 0.07, and RMR = 0.25. Chi-square difference test between unconstrained (χ2 = 190.8, df = 48) and constrained (χ2 = 196.2, df = 57) structural models for two groups of data collection model and we found invariant with *p* = 0.79. Therefore, groups are not different at model level, however they may be different at the path level. MIMIC model of the latent factors of aMBI burnout scale (EE, DP, and PA) was performed for different methods of data collection (online or paper), we found that none of the latent variables yield statistically significant coefficients, which means that there was no difference between the two methods of data collection as follows: Emotional Exhaustion (Regression Weight Estimate = −0.148, Standardized Error (S.E.) = 0.156, C.R. = −0.949, *p* = 0.343), Depersonalization (Regression Weight Estimate=0.0.77, Standardized Error (S.E.) = 0.139, C.R. = 0.553, *p* = 0.580), and Personal accomplishment (Regression Weight Estimate = −0.071, Standardized Error (S.E.) = 0.076, C.R. = −0.939, *p* = 0.348). Figure 1B shows the MIMIC model using paper-online variable as a differential factor.

**Table 2:** Parameter and standard error estimates for the model of Figure 1A.

**Model Parameters**	**Standardized estimate**	**Unstandardized estimate**	**Standard error**	***p*-value**
**Loadings/effects on aMBI**				
I feel emotionally drained from my work	0.77	1.00^a^		
I feel fatigued when I get up in the morning and have to face another day on the job	0.73	0.91	0.05	<0.001^**^
Working with people all day is really a strain for me	0.68	0.91	0.06	<0.001^**^
I feel I treat some patients as if they were impersonal objects	0.57	1.00^a^		
I've become more callous toward people since I took this job	0.78	1.33	0.11	<0.001^**^
I don't really care what happens to some patients	0.54	0.91	0.09	<0.001^**^
I deal very effectively with the problems of my patients	0.38	1.00^a^		
I feel I'm positively influencing other people's lives through my work	0.55	1.68	0.29	<0.001^**^
I feel exhilarated after working closely with my patients	0.56	1.89	0.32	<0.001^**^
**Covariances**				
Emotional exhaustion < -> depersonalization	1.76		0.18	<0.001^**^
Depersonalization < -> personal accomplishment	0.43		0.08	<0.001^**^
Emotional exhaustion < -> personal accomplishment	0.54		0.09	<0.001^**^

“a” mean for the Figure 1A.

** *Significant at (p < 0.001)*.

### Burnout Results Using the Abbreviated Maslach Burnout Inventory

Of the study participants, 357 (67.1%) reported experiencing high emotional exhaustion (EE Score ≥ 10), while 252 (47.4%) reported experiencing depersonalization (DP score ≥ 10), and only 121 (22.7%) reported a lower sense of personal accomplishment (PA score ≤ 10). The mean score of emotional exhaustion was 11.3 (SD = 4.8). For depersonalization, the mean score was 8.5 (SD = 5.1), while for personal accomplishment mean score was 12.7 (SD = 3.7).

Table 3 presents the scores for the entire scale and its subscales. We found a statistically significant association between emotional exhaustion and gender, years of work experience, department, and living in a conflict area (*p* < 0.05). When we compared depersonalization with study characteristics, we found a statistically significant association between gender, age, department, internal displacement, and verbal abuse (*p* < 0.05). However, for personal accomplishment [Decreased personal accomplishment (≤ 10) and Mod-High personal accomplishment (>10)], we did not identify a statistically significant association with other study variables. A comprehensive comparison between participants characteristics and subscales of burnout can be found in Supplementary Material.

**Table 3:** Scores of participants according to the abbreviated Maslach Burnout Inventory.

**Subscale**	**Mean ± SD**	**Range (min–max)**	**Percentile**		
			**25th**	**50th**	**75th**
Emotional exhaustion	11.25 ± 4.81	0–18	8	12	15
Depersonalization	8.5 ± 5.06	0–18	4.25	9	12.75
Decreased personal accomplishment	12.74 ± 3.74	0–18	11	13.5	15
Emotional exhaustion + depersonalization	19.76 ± 8.99	0–36	12	21	27

*SD, standard deviation*.

#### Emotional Exhaustion

**Figure 2:**
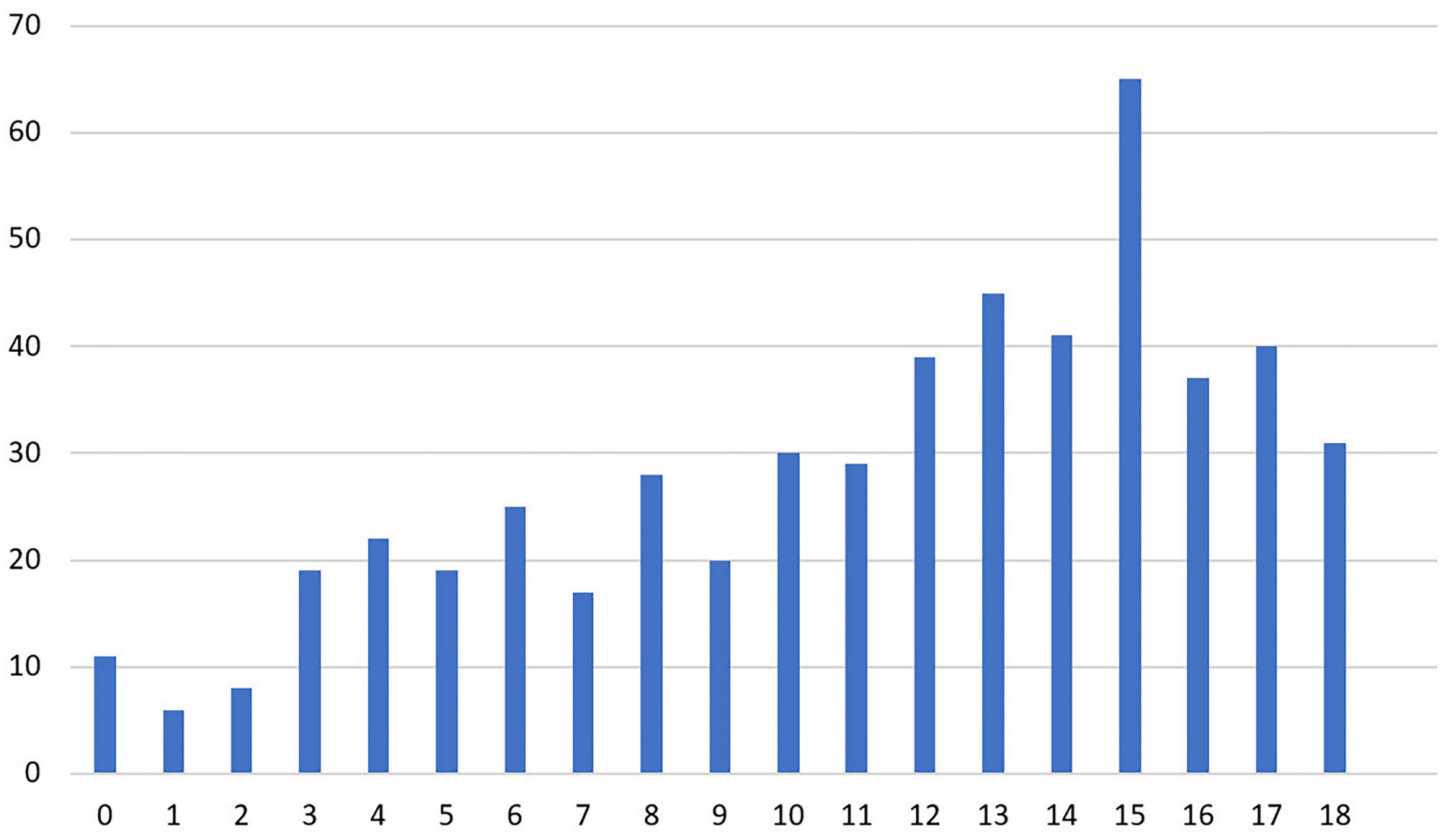
Number of physician responses for the emotional exhaustion subscale of the abbreviated Maslach Burnout Inventory.

Three hundred and fifty-seven (67.1%) participants scored 10 or higher for EE, indicating a higher risk of burnout syndrome (see Figure 2). A Spearman's rank-order correlation test identified a positive correlation between gender and EE score [rs (530) = 0.099; *p* = 0.022]. There was a negative correlation between age and EE score [rs (530) = −0.151; *p* < 0.001]. A significant negative correlation was found regarding years of experience [rs (530) = −0.118; *p* = 0.007], while a significant positive correlation was identified between department type and EE score [rs (530) = 0.113; *p* = 0.009], living in conflict area [rs (530) = 0.13; *p* = 0.003], feeling stigmatized [rs (530) = 0.174; *p* ≤ 0.001], and working hours per week [rs (530) = 0.125; *p* = 0.004]. There was no statistically significant correlation between EE score and marital status, living conditions, employment status, smoking, illicit drug use, internal displacement, verbal abuse, physical abuse, and number of shifts per month (*p* > 0.05).

#### Depersonalization

**Figure 3:**
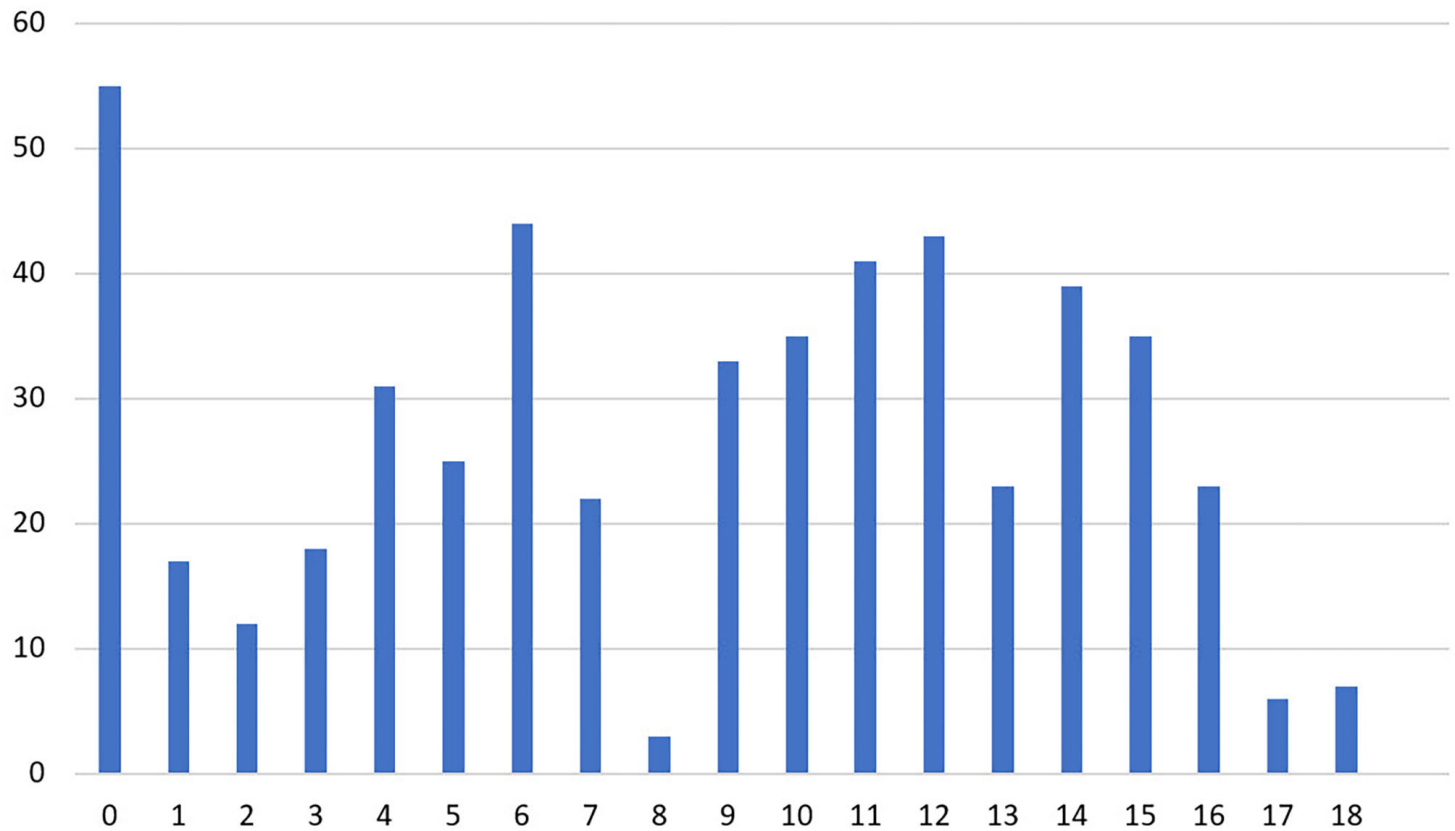
Number of physician responses for the depersonalization subscale of the abbreviated Maslach Burnout Inventory.

Two hundred and fifty-two (47.4%) participants scored 10 or higher for DP, indicating a higher risk of burnout syndrome (Figure 3). A Spearman's rank-order test showed a positive correlation between gender and DP score [rs (530) = 0.129; *p* = 0.003], department type [rs (530) = 0.105; *p* = 0.015], internal displacement [rs (530) = 0.119; *p* = 0.006], and living in conflict area [rs (530) = 0.09; *p* = 0.038].

However, there was no statistically significant correlation between DP score and age, marital status, living conditions, employment status, feeling stigmatized, smoking, illicit drug use, verbal abuse, physical abuse, working hours per week, and number of shifts per month (*p* > 0.05). Two hundred and thirty-six (44.4%) scored ten or more on both EE and DP, representing a higher risk of burnout syndrome.

#### Personal Accomplishment

**Figure 4:**
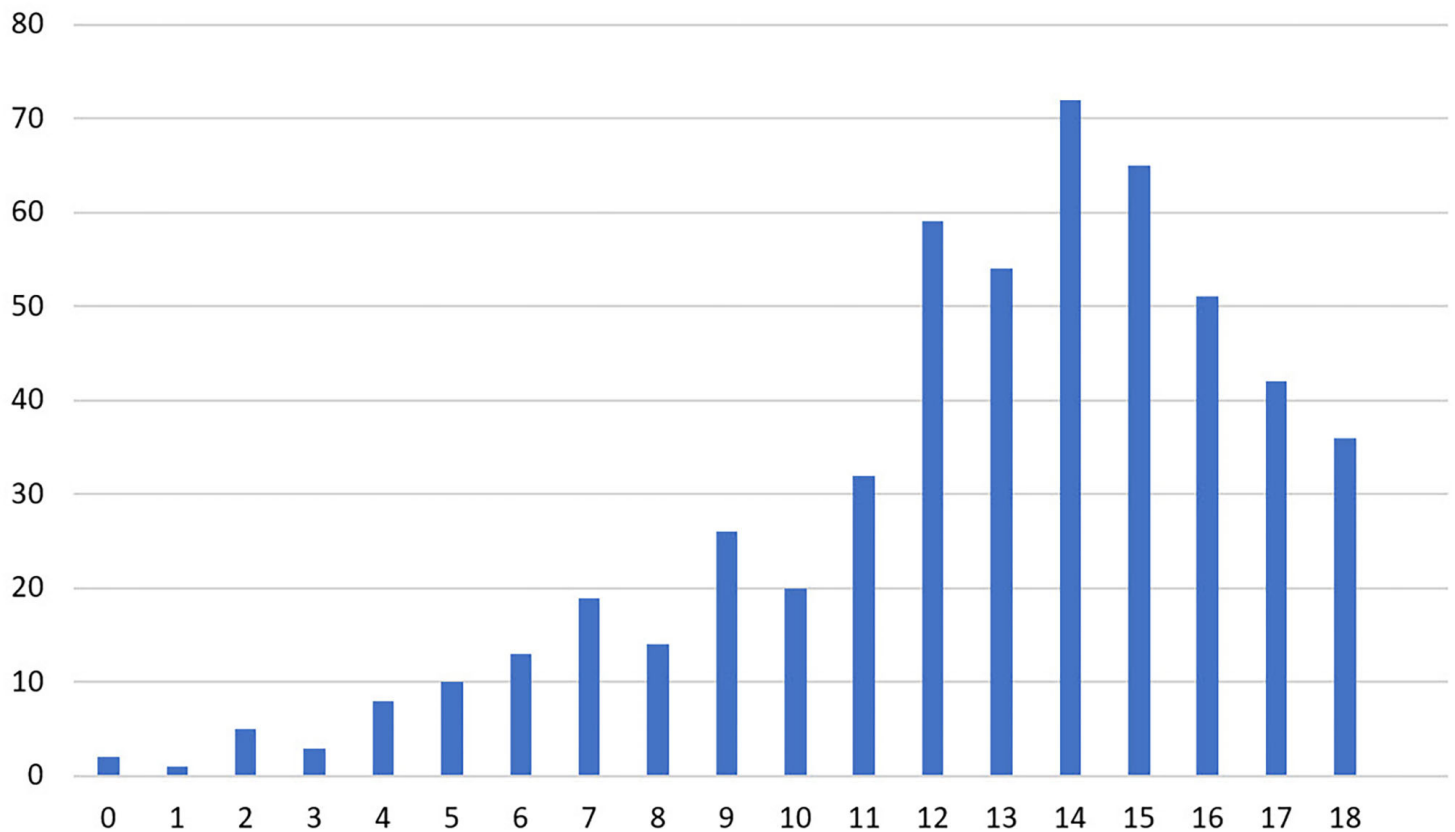
Number of physician responses for the personal accomplishment subscale of the abbreviated Maslach Burnout Inventory.

One hundred and twenty-one (22.7%) participants scored ≤ 10 for PA, indicating a higher risk of burnout syndrome (Figure 4). A Spearman's rank-order correlation test indicated that none of the study subject characteristics were correlated with PA score such as gender, marital status, age, years of work experience, department, living conditions, employment, feeling stigmatized, smoking, illicit drug use, internal displacement, living in a conflict area, verbal abuse, physical abuse, working hours per week, and number of night shifts per month (*p* > 0.05).

## Discussion

This study aimed to assess burnout among healthcare workers in departments which are high-risk for COVID-19 in Libya, which is currently experiencing a civil war. To our knowledge, the present study is the first to examine burnout syndrome during COVID-19 in a civil war setting.

The present study demonstrated a high prevalence of anxiety and depression among physicians during the COVID-19 pandemic amidst the civil war. The study provided a justified sample size of 532 physicians working on the frontlines of the pandemic in March and April of 2020. The response rate and data completion were in good range. Risk of burnout scores reported by participants were 67.1% for emotional exhaustion, 47.4% for depersonalization, and 22.7% for lower personal accomplishment. However, 44.4% have both emotional exhaustion and depersonalization.

In our study, gender (35) was associated with both high EE and high DP. However, it was not associated with a low PA. In terms of age, being 35 years or older was associated with high depersonalization. Marital status was not associated with high levels of burnout. High-risk departments for COVID-19 were significantly associated with high EE and DP; specifically, those in the surgical department, emergency department, and intensive care units were more likely to have high EE and DP. Personal accomplishment was not associated with the department or professional specialty. Employment sector, i.e., government or private, was not found to be associated with burnout. In addition, fear of COVID-19 infection was associated with higher EE and DP.

Years of work experience was statistically associated with personal accomplishment. Our findings indicated that those with higher personal accomplishment scores were those with less working experience, and those with more experience felt less accomplishment. Smoking and illicit drug use were not associated with EE or DP. Feelings of stigmatization due to COVID-19 was associated with high scores in DP. In addition, verbal abuse was associated with DP only. Internal displacement and verbal abuse were associated with higher risk of DP. Living in conflict area was associated with higher risk of EE.

We observed that burnout prevalence among Libyan physicians was higher than in other countries. A study on radiology residents in the USA found that high EE, high DP, and low PA scores were reported by 37, 48, and 50% of participants, respectively (42). A further study conducted among Iranian healthcare workers reported participant scores of 12.3, 5.3, and 43% for high EE, high DP, and low PA, respectively (43). In Nigeria, a study conducted among residency training physicians found that 45.6% of physicians had high EE, 57.8% had high DP, and 61.8% had low PA. These findings were similar to those of the present study. However, a systematic review and meta-analysis of 4,664 medical residents found that the overall prevalence of burnout was 35.7% for surgical staff, and critical care workers had a higher prevalence of 40.8% (44). Additionally, a study conducted in Malaysia showed a burnout prevalence of 26.5% among junior doctors (45), and another study in Saudi Arabia indicated that 25.2% of physicians can be classified as having burnout (35). This wide variation is explained by socioeconomic and cultural differences, as well as the differences in healthcare infrastructure among these countries. However, a study in the United States found that about 50% of physicians suffer from burnout, which is similar to our results (46).

Burnout is known to be highly prevalent among physicians due to the psychologically demanding nature of the profession. Physicians are also exposed to a high level of socioeconomic pressure that may lead to burnout. Burnout has been found to be a risk factor for training attrition, suicide, and low quality of life. It has further been linked with sleep deprivation, family issues, and feeling overwhelmed with tasks and paperwork (20). In addition, burnout was associated with a high level of medical errors and a decreased level of patient care (47). Therefore, interventions aimed at reducing the stress levels of physicians are needed to improve well-being and quality of life among this population (48). For example, coaching for female physicians who are undergoing strain and experiencing stress about starting a family, and need external support from general practitioners, have been suggested in previous research (49).

There are a number of contributing factors for the increased level of burnout among Libyan physicians found in this study. First, due to the country's economic crisis, these healthcare workers are irregularly compensated and suffer socioeconomic hardships. Second, physicians are subject to a high level of abuse by patients and militias (50). The present study found that the prevalence of verbal abuse and physical abuse is 57 and 17.5%, respectively. Additionally, the civil war has caused displacement among physicians; 32.5% of physicians have left their homes due to the conflict. This situation places more pressure on these healthcare professionals, who fear their homes being destroyed or taken over by militias (51). Finally, they have concerns regarding the COVID-19 pandemic. The physicians fear being infected and infecting their families. They also have concerns about the shortage of treatment supplies and personal protective equipment (52, 53).

The present study has several limitations. First, because of the observational study design, we were unable to determine causation or demonstrate strong relationships between variables. Therefore, larger studies are needed to examine predictive factors and to focus on other contributing factors that were not included in the present study. Second, the present study was conducted in one country where physicians face multiple stressors, including COVID-19, civil war, financial crisis, and a scarcity of mental health centers, which can explain the high level of burnout we observed. In addition, we believe that the fear of stigmatization may have resulted in response bias. Another limitation is that there is no standard definition of burnout. Although the aMBI is a validated tool that can detect and screen those who are at high risk of burnout, some studies have discussed the overestimation of the tool's effectiveness (32, 54, 55).

This study highlights the importance of addressing burnout among healthcare workers. Issues of burnout should be prioritized by authorities. This study identified a high demand for physician support interventions such as social support programs, financial support, and increased security measures in hospitals to prevent and decrease abuse. Furthermore, there is a need to recognize external contributing factors and the impact they have on physician's lives, such as internal displacement and living in conflict areas. Thus, the government should provide support in these areas to prevent humanitarian crises.

In conclusion, the rising prevalence of mental disorders among physicians and inadequate availability of healthcare facilities during the COVID-19 pandemic and civil war has demonstrated the need for healthcare policies to address the well-being of healthcare workers to decrease the risk of medical negligence, deteriorating mental health, and suicide.

## Data Availability Statement

The raw data supporting the conclusions of this article will be made available by the authors, without undue reservation.

## Ethics Statement

The studies involving human participants were reviewed and approved by Bioethics Committee of the Biotechnology Research Center in Libya. The patients/participants provided their written informed consent to participate in this study.

## Author Contributions

MElh analyzed and interpreted the data, supervised the project, and wrote the first draft of the manuscript. All authors contributed to the study design and data collection. All authors have read and approved the final manuscript.

## Conflict of Interest

The authors declare that the research was conducted in the absence of any commercial or financial relationships that could be construed as a potential conflict of interest.

## References

[1] Zhou P Yang XL Wang XG Hu B Zhang L Zhang W. A pneumonia outbreak associated with a new coronavirus of probable bat origin. Nature. (2020) 579:270–3. 10.1038/s41586-020-2012-7 32015507 PMC7095418

[2] World Health Organization Director-General's Remarks at the Media Briefing on 2019-nCoV on 11 February 2020. WHO (2020). Available online at: https://www.who.int/director-general/speeches/detail/who-director-general-s-remarks-at-the-media-briefing-on-2019-ncov-on-11-february-2020

[3] Dong E Du H Gardner L. An interactive web-based dashboard to track COVID-19 in real time. Lancet Infect Dis. (2020) 20:533–4. 10.1016/S1473-3099(20)30120-1 32087114 PMC7159018

[4] World Health Organization WHO Director-General's Opening Remarks at the Media Briefing on COVID-19 - 22 April 2020. (2020). Available online at: https://www.who.int/dg/speeches/detail/who-director-general-s-opening-remarks-at-the-media-briefing-on-covid-19-22-april-2020 (accessed May 1, 2020).

[5] Elhadi M Momen AA Ali Senussi Abdulhadi OM. A COVID-19 case in Libya acquired in Saudi Arabia. Travel Med Infect Dis. (2020) 37:101705. 10.1016/j.tmaid.2020.101705 32360409 PMC7252057

[6] Lai J Ma S Wang Y Cai Z Hu J Wei N. Factors Associated with mental health outcomes among health care workers exposed to coronavirus disease 2019. JAMA Network Open. (2020) 3:e203976. 10.1001/jamanetworkopen.2020.3976 32202646 PMC7090843

[7] Wang D Hu B Hu C Zhu F Liu X Zhang J. Clinical characteristics of 138 hospitalized patients with 2019 novel coronavirus-infected pneumonia in Wuhan, China. JAMA. (2020) 323:1061–9. 10.1001/jama.2020.1585 32031570 PMC7042881

[8] Xiang YT Jin Y Cheung T. Joint international collaboration to combat mental health challenges during the coronavirus disease 2019 pandemic. JAMA Psychiatry. (2020) 77:989–90. 10.1001/jamapsychiatry.2020.1057 32275289

[9] Tan BYQ Chew NWS Lee GKH Jing M Goh Y Yeo LLL. Psychological Impact of the COVID-19 Pandemic on Health Care Workers in Singapore. Ann Intern Med. (2020) 173:317–20. 10.7326/M20-1083 32251513 PMC7143149

[10] Freudenberger HJ Staff burn-out. J Soc Issues. (1974) 30:159–65. 10.1111/j.1540-4560.1974.tb00706.x

[11] Ishak W Nikravesh R Lederer S Perry R Ogunyemi D Bernstein C. Burnout in medical students: a systematic review. Clin Teach. (2013) 10:242–5. 10.1111/tct.12014 23834570

[12] Ishak WW Lederer S Mandili C Nikravesh R Seligman L Vasa M. Burnout during residency training: a literature review. J Grad Med Educ. (2009) 1:236–42. 10.4300/JGME-D-09-00054.1 21975985 PMC2931238

[13] Bianchi R Schonfeld IS Laurent E. Is it time to consider the burnout syndrome a distinct illness? Front Public Health. (2015) 3:158. 10.3389/fpubh.2015.00158 26106593 PMC4459038

[14] Bridgeman PJ Bridgeman MB Barone J. Burnout syndrome among healthcare professionals. Am J Health Syst Pharm. (2018) 75:147–52. 10.2146/ajhp170460 29183877

[15] Halbesleben JR Rathert C. Linking physician burnout and patient outcomes: exploring the dyadic relationship between physicians and patients. Health Care Manage Rev. (2008) 33:29–39. 10.1097/01.HMR.0000304493.87898.72 18091442

[16] Hewitt DB Ellis RJ Chung JW Cheung EO Moskowitz JT Huang R. Association of surgical resident wellness with medical errors and patient outcomes. Ann Surg. (2020). 10.1097/SLA.0000000000003909. [Epub ahead of print]. 32282379

[17] Martin F Poyen D Bouderlique E Gouvernet J Rivet B Disdier P. Depression and burnout in hospital health care professionals. Int J Occup Environ Health. (1997) 3:204–9. 10.1179/oeh.1997.3.3.204 9891120

[18] Dyrbye LN Thomas MR Massie FS Power DV Eacker A Harper W. Burnout and suicidal ideation among U.S. medical students. Ann Intern Med. (2008) 149:334–41. 10.7326/0003-4819-149-5-200809020-00008 18765703

[19] Rothenberger DA. Physician burnout and well-being: a systematic review and framework for action. Dis Colon Rectum. (2017) 60:567–76. 10.1097/DCR.0000000000000844 28481850

[20] Stehman CR Testo Z Gershaw RS Kellogg AR Burnout, drop out, suicide: physician loss in emergency medicine, part i. West J Emerg Med. (2019) 20:485–94. 10.5811/westjem.2019.4.40970 31123550 PMC6526882

[21] Melamed S Shirom A Toker S Berliner S Shapira I. Burnout and risk of cardiovascular disease: evidence, possible causal paths, and promising research directions. Psychol Bull. (2006) 132:327–53. 10.1037/0033-2909.132.3.327 16719565

[22] Lo EV Wei YH Hwang BF. Association between occupational burnout and heart rate variability: a pilot study in a high-tech company in Taiwan. Medicine. (2020) 99:e18630. 10.1097/MD.0000000000018630 31914045 PMC6959968

[23] Toker S Shirom A Shapira I Berliner S Melamed S. The association between burnout, depression, anxiety, and inflammation biomarkers: C-reactive protein and fibrinogen in men and women. J Occup Health Psychol. (2005) 10:344–62. 10.1037/1076-8998.10.4.344 16248685

[24] Summerfield D. War and mental health: a brief overview. BMJ. (2000) 321:232–5. 10.1136/bmj.321.7255.232 10903662 PMC1118225

[25] Levy BS Sidel VW. Health effects of combat: a life-course perspective. Annu Rev Public Health. (2009) 30:123–36. 10.1146/annurev.publhealth.031308.100147 18925871

[26] Okasha A Karam E Okasha T. Mental health services in the Arab world. World Psychiatry. (2012) 11:52–4. 10.1016/j.wpsyc.2012.01.008 22295010 PMC3266748

[27] Rhouma AH Husain N Gire N Chaudhry IB. Mental health services in Libya. BJPsych Int. (2016) 13:70–1. 10.1192/S2056474000001288 29093908 PMC5618881

[28] Maslach C Jackson SE Leiter MP Schaufeli WB Schwab RL. Maslach Burnout Inventory. Palo Alto, CA: Consulting Psychologists Press (1986).

[29] Mcmanus IC Keeling A Paice E. Stress, burnout and doctors' attitudes to work are determined by personality and learning style: a twelve year longitudinal study of UK medical graduates. BMC Med. (2004) 2:29. 10.1186/1741-7015-2-29 15317650 PMC516448

[30] Maslach C Leiter MP. Early predictors of job burnout and engagement. J Appl Psychol. (2008) 93:498–512. 10.1037/0021-9010.93.3.498 18457483

[31] Mcclafferty H Brown OW. Physician health and wellness. Pediatrics. (2014) 134:830–5. 10.1542/peds.2014-2278 25266440

[32] Mcmanus IC Winder BC Gordon D. The causal links between stress and burnout in a longitudinal study of UK doctors. Lancet. (2002) 359:2089–90. 10.1016/S0140-6736(02)08915-8 12086767

[33] Langade D Modi PD Sidhwa YF Hishikar NA Gharpure AS Wankhade K. Burnout syndrome among medical practitioners across India: a questionnaire-based survey. Cureus. (2016) 8:e771. 10.7759/cureus.771 27833826 PMC5101402

[34] Waddimba AC Scribani M Nieves MA Krupa N May JJ Jenkins P. Validation of single-item screening measures for provider burnout in a rural health care network. Eval Health Prof. (2016) 39:215–25. 10.1177/0163278715573866 25716107

[35] Bawakid K Abdulrashid O Mandoura N Shah HBU Ibrahim A Akkad NM. Burnout of physicians working in primary health care centers under Ministry of Health Jeddah, Saudi Arabia. Cureus. (2017) 9:e1877. 10.7759/cureus.1877 29383297 PMC5784861

[36] Lebares CC Guvva EV Ascher NL O'sullivan PS Harris HW Epel ES. Burnout and stress among US surgery residents: psychological distress and resilience. J Am Coll Surg. (2018) 226:80–90. 10.1016/j.jamcollsurg.2017.10.010 29107117

[37] Riley MR Mohr DC Waddimba AC. The reliability and validity of three-item screening measures for burnout: evidence from group-employed health care practitioners in upstate New York. Stress Health. (2018) 34:187–93. 10.1002/smi.2762 28524379

[38] Beckstead JW. Confirmatory factor analysis of the Maslach Burnout Inventory among Florida nurses. Int J Nurs Stud. (2002) 39:785–92. 10.1016/S0020-7489(02)00012-3 12379296

[39] Browne MW Cudeck R Alternative ways of assessing model fit. Sociol Methods Res. (1992) 21:230–58. 10.1177/0049124192021002005

[40] Bentler PM. Comparative fit indexes in structural models. Psychol Bull. (1990) 107:238–46. 10.1037/0033-2909.107.2.238 2320703

[41] Hu LT Bentler PM Cutoff criteria for fit indexes in covariance structure analysis: conventional criteria versus new alternatives. Struct Equ Modeling. (1999) 6:1–55. 10.1080/10705519909540118

[42] Guenette JP Smith SE. Burnout: prevalence and associated factors among radiology residents in New England with comparison against United States resident physicians in other specialties. AJR Am J Roentgenol. (2017) 209:136–41. 10.2214/AJR.16.17541 28639920

[43] Malakouti SK Nojomi M Salehi M Bijari B. Job stress and burnout syndrome in a sample of rural health workers, behvarzes, in Tehran, Iran. Iran J Psychiatry. (2011) 6:70–4. 22952525 PMC3395939

[44] Rodrigues H Cobucci R Oliveira A Cabral JV Medeiros L Gurgel K. Burnout syndrome among medical residents: a systematic review and meta-analysis. PLoS ONE. (2018) 13:e0206840. 10.1371/journal.pone.0206840 30418984 PMC6231624

[45] As Z Zainal NZ Exploring burnout among Malaysian junior doctors using the abbreviated maslach burnout inventory. Malaysian J Psychiatry. (2015). 24. [Epub ahead of print].

[46] Shanafelt TD Dyrbye LN West CP. Addressing physician burnout: the way forward. JAMA. (2017) 317:901–2. 10.1001/jama.2017.0076 28196201

[47] Lu DW Dresden S Mccloskey C Branzetti J Gisondi MA. Impact of burnout on self-reported patient care among emergency physicians. West J Emerg Med. (2015) 16:996–1001. 10.5811/westjem.2015.9.27945 26759643 PMC4703144

[48] West CP Dyrbye LN Erwin PJ Shanafelt TD. Interventions to prevent and reduce physician burnout: a systematic review and meta-analysis. Lancet. (2016) 388:2272–81. 10.1016/S0140-6736(16)31279-X 27692469

[49] Petek D Gajsek T Petek Ster M. Work-family balance by women GP specialist trainees in Slovenia: a qualitative study. BMC Med Educ. (2016) 16:31. 10.1186/s12909-016-0551-2 26821533 PMC4730732

[50] Elhadi M Khaled A Malek AB El-Azhari AE Gwea AZ Zaid A. Prevalence of anxiety and depressive symptoms among emergency physicians in Libya after civil war: a cross-sectional study. BMJ Open. (2020) 10:e039382. 10.1136/bmjopen-2020-039382 32859667 PMC7454180

[51] Elhadi M Msherghi A Elgzairi M Alhashimi A Bouhuwaish A Biala M. Psychological status of healthcare workers during the civil war and COVID-19 pandemic: a cross-sectional study. J Psychosom Res. (2020) 137:110221. 10.1016/j.jpsychores.2020.110221 32827801 PMC7428743

[52] Joob B Wiwanitkit V. Medical personnel, COVID-19 and emotional impact. Psychiatry Res. (2020) 288:112952. 10.1016/j.psychres.2020.112952 32335465 PMC7152901

[53] Lima CKT Carvalho PMM Lima I Nunes J Saraiva JS De Souza RI. The emotional impact of coronavirus 2019-nCoV (new coronavirus disease). Psychiatry Res. (2020) 287:112915. 10.1016/j.psychres.2020.112915 32199182 PMC7195292

[54] Panagioti M Geraghty K Johnson J Zhou A Panagopoulou E Chew-Graham C. Association between physician burnout and patient safety, professionalism, and patient satisfaction: a systematic review and meta-analysis. JAMA Intern Med. (2018) 178:1317–30. 10.1001/jamainternmed.2018.3713 30193239 PMC6233757

[55] Low ZX Yeo KA Sharma VK Leung GK Mcintyre RS Guerrero A. Prevalence of burnout in medical and surgical residents: a meta-analysis. Int J Environ Res Public Health. (2019) 16. 10.3390/ijerph16091479 31027333 PMC6539366

