# Supplementary material for: Burnout Syndrome Among Hospital Healthcare Workers During the COVID-19 Pandemic and Civil War: A Cross-Sectional Study
Source: Front Psychiatry. 2020 Dec 11;11:579563. doi: 10.3389/fpsyt.2020.579563 (PMC7759513; doi:10.3389/fpsyt.2020.579563)
Supplement: Supplementary file 1 [file Data_Sheet_1.docx]

# Abbreviated Maslach burnout inventory comparison with study characteristics

*Supplementary Table 1. Men vs women*

|  | **Men n (%)** | **Women n (%)** | **Total** |
| --- | --- | --- | --- |
| **High Emotional exhaustion (≥10)** | 209 (71.1) | 148 (62.2) | 357 |
| **Low Emotional exhaustion (<10)** | 85 (28.9) | 90 (37.8) | 175 |
| **Total** | 294 | 238 | 532 |
| **Phi (φ)** |  |  | 0.094 |
| **p (Fisher’s exact test)** |  |  | **0.033*** |
|  |  |  |  |
| **High Depersonalization (≥10)** | 152 (51.7) | 100 (42) | 252 |
| **Low Depersonalization (<10)** | 142 (48.3) | 138 (58) | 280 |
| **Total** | 294 | 238 | 532 |
| **Phi (φ)** |  |  | 0.096 |
| **p (Fisher’s exact test)** |  |  | **0.029*** |
|  |  |  |  |
| **Decreased personal accomplishment (≤10)** | 58 (19.7) | 63 (26.5) | 121 |
| **Mod-High personal accomplishment (>10)** | 236 (80.3) | 175 (73.5) | 411 |
| **Total** | 294 | 238 | 532 |
| **Phi (φ)** |  |  | -0.08 |
| **p (Fisher’s exact test)** |  |  | **0.77** |

*Supplementary Table 2. Age < 35 vs* **≥** *35 years*

|  | **< 35 n (%)** | **≥ 35 n (%)** | **Total** |
| --- | --- | --- | --- |
| **High Emotional exhaustion (≥10)** | 272 (69.4) | 85 (60.7) | 357 |
| **Low Emotional exhaustion (<10)** | 120 (30.6) | 55 (39.3) | 175 |
| **Total** | 392 | 140 | 532 |
| **Phi (φ)** |  |  | -0.081 |
| **p (Fisher’s exact test)** |  |  | **0.075** |
|  |  |  |  |
| **High Depersonalization (≥10)** | 197 (50.3) | 55 (39.3) | 252 |
| **Low Depersonalization (<10)** | 195 (49.7) | 85 (60.7) | 280 |
| **Total** | 392 | 140 | 532 |
|  |  |  | -0.097 |
| **p (Fisher’s exact test)** |  |  | **0.03*** |
|  |  |  |  |
| **Decreased personal accomplishment (≤10)** | 83 (21.2) | 38 (27.1) | 121 |
| **Mod-High personal accomplishment (>10)** | 309 (78.8) | 102 (72.9) | 411 |
| **Total** | 392 | 140 | 532 |
| **Phi (φ)** |  |  | 0.063 |
| **p (Fisher’s exact test)** |  |  | **0.159** |

*Supplementary Table 3. Marital status*

|  | **Not-married n (%)** | **Married n (%)** | **Total** |
| --- | --- | --- | --- |
| **High Emotional exhaustion (≥10)** | 213 (68.9) | 144 (64.6) | 357 |
| **Low Emotional exhaustion (<10)** | 96 (31.1) | 79 (34.4) | 175 |
| **Total** | 309 | 223 | 532 |
| **Phi (φ)** |  |  | -0.046 |
| **p (Fisher’s exact test)** |  |  | **0.305** |
|  |  |  |  |
| **High Depersonalization (≥10)** | 150 (48.5) | 102 (45.7) | 252 |
| **Low Depersonalization (<10)** | 159 (51.5) | 121 (54.3) | 280 |
| **Total** | 309 | 223 | 532 |
| **Phi (φ)** |  |  | -0.028 |
| **p (Fisher’s exact test)** |  |  | **0.539** |
|  |  |  |  |
| **Decreased personal accomplishment (≤10)** | 66 (21.4) | 55 (24.7) | 121 |
| **Mod-High personal accomplishment (>10)** | 243 (78.6) | 168 (75.3) | 411 |
| **Total** | 309 | 223 | 532 |
| **Phi (φ)** |  |  | 0.039 |
| **p (Fisher’s exact test)** |  |  | **0.402** |

*Supplementary Table 4. Living status*

|  | **With Family n (%)** | **Alone n (%)** | **Total** |
| --- | --- | --- | --- |
| **High Emotional exhaustion (≥10)** | 236 (67.8) | 121 (65.8) | 357 |
| **Low Emotional exhaustion (<10)** | 112 (32.2) | 63 (34.2) | 175 |
| **Total** | 348 | 184 | 532 |
| **Phi (φ)** |  |  | -0.021 |
| **p (Fisher’s exact test)** |  |  | **0.305** |
|  |  |  |  |
| **High Depersonalization (≥10)** | 164 (47.1) | 88 (47.8) | 252 |
| **Low Depersonalization (<10)** | 184 (52.9) | 96 (52.2) | 280 |
| **Total** | 348 | 184 | 532 |
| **Phi (φ)** |  |  | 0.007 |
| **p (Fisher’s exact test)** |  |  | **0.927** |
|  |  |  |  |
| **Decreased personal accomplishment (≤10)** | 75 (21.6) | 46 (25) | 121 |
| **Mod-High personal accomplishment (>10)** | 273 (78.4) | 138 (75) | 411 |
| **Total** | 348 | 184 | 532 |
| **Phi (φ)** |  |  | 0.039 |
| **p (Fisher’s exact test)** |  |  | **0.385** |

*Supplementary Table 5. Employment status*

|  | **Governmental n (%)** | **Private n (%)** | **Both n (%)** | **Total** |
| --- | --- | --- | --- | --- |
| **High Emotional exhaustion (≥10)** | 162 (66.4) | 45 (69.2) | 150 (67.3) | 357 |
| **Low Emotional exhaustion (<10)** | 82 (33.6) | 20 (30.8) | 73 (32.7) | 175 |
| **Total** | 244 | 65 | 223 | 532 |
| **Cramer's V** |  |  |  | 0.019 |
| **p (Chi-square test)** |  |  |  | **0.909** |
|  |  |  |  |  |
| **High Depersonalization (≥10)** | 112 (45.9) | 31 (47.7) | 109 (48.9) | 252 |
| **Low Depersonalization (<10)** | 132 (54.1) | 34 (52.3) | 114 (51.1) | 280 |
| **Total** | 244 | 65 | 223 | 532 |
| **Cramer's V** |  |  |  | 0.028 |
| **p (Chi-square test)** |  |  |  | **0.812** |
|  |  |  |  |  |
| **Decreased personal accomplishment (≤10)** | 59 (24.2) | 17 (26.2) | 45 (20.2) | 121 |
| **Mod-High personal accomplishment (>10)** | 185 (75.8) | 48 (73.8) | 178 (79.8) | 411 |
| **Total** | 244 | 65 | 223 | 532 |
| **Cramer's V** |  |  |  | 0.054 |
| **p (Chi-square test)** |  |  |  | **0.460** |

*Supplementary Table 6. Years of experience*

|  | **< 3 years n (%)** | **3-5 years n (%)** | **5-15 years n (%)** | **> 15 years n (%)** | **Total** |
| --- | --- | --- | --- | --- | --- |
| **High Emotional exhaustion (≥10)** | 167 (72.3) | 67 (60.4) | 99 (69.2) | 24 (51.1) | 357 |
| **Low Emotional exhaustion (<10)** | 64 (27.7) | 44 (39.6) | 44 (30.8) | 23 (48.9) | 175 |
| **Total** | 231 | 111 | 143 | 47 | 532 |
| **Cramer's V** |  |  |  |  | 0.143 |
| **p (Chi-square test)** |  |  |  |  | **0.012*** |
|  |  |  |  |  |  |
| **High Depersonalization (≥10)** | 118 (51.1) | 51 (45.9) | 66 (46.2) | 17 (36.2) | 252 |
| **Low Depersonalization (<10)** | 113 (48.9) | 60 (54.1) | 77 (53.8) | 30 (63.8) | 280 |
| **Total** | 231 | 111 | 143 | 47 | 532 |
| **Cramer's V** |  |  |  |  | 0.085 |
| **p (Chi-square test)** |  |  |  |  | **0.282** |
|  |  |  |  |  |  |
| **Decreased personal accomplishment (≤10)** | 44 (19) | 35 (31.5) | 29 (20.3) | 13 (27.7) | 121 |
| **Mod-High personal accomplishment (>10)** | 187 (81) | 76 (68.5) | 114 (79.7) | 34 (72.3) | 411 |
| **Total** | 231 | 111 | 143 | 47 | 532 |
| **Cramer's V** |  |  |  |  | 0.121 |
| **p (Chi-square test)** |  |  |  |  | **0.050** |

*Supplementary Table 7. Department*

|  | **Internal Medicine n (%)** | **Surgery n (%)** | **Emergency n (%)** | **Intensive Care n (%)** | **Total** |
| --- | --- | --- | --- | --- | --- |
| **High Emotional exhaustion (≥10)** | 126 (56.5) | 101 (75.4) | 77 (69.4) | 53 (82.8) | 357 |
| **Low Emotional exhaustion (<10)** | 97 (43.5) | 33 (24.6) | 34 (30.6) | 11 (17.2) | 175 |
| **Total** | 223 | 134 | 111 | 64 | 532 |
| **Cramer's V** |  |  |  |  | 0.21 |
| **p (Chi-square test)** |  |  |  |  | **<0.001*** |
|  |  |  |  |  |  |
| **High Depersonalization (≥10)** | 92 (41.3) | 73 (54.5) | 59 (53.2) | 28 (43.8) | 252 |
| **Low Depersonalization (<10)** | 131 (58.7) | 61 (45.5) | 52 (46.8) | 36 (56.2) | 280 |
| **Total** | 223 | 134 | 111 | 64 | 532 |
| **Cramer's V** |  |  |  |  | 0.12 |
| **p (Chi-square test)** |  |  |  |  | **0.048*** |
|  |  |  |  |  |  |
| **Decreased personal accomplishment (≤10)** | 56 (25.1) | 22 (16.4) | 27 (24.3) | 16 (25) | 121 |
| **Mod-High personal accomplishment (>10)** | 167 (74.9) | 112 (83.6) | 84 (75.7) | 48 (75) | 411 |
| **Total** | 223 | 134 | 111 | 64 | 532 |
| **Cramer's V** |  |  |  |  | 0.88 |
| **p (Chi-square test)** |  |  |  |  | **0.250** |

*Supplementary Table 8. Internal displacement*

|  | **No n (%)** | **Yes n (%)** | **Total** |
| --- | --- | --- | --- |
| **High Emotional exhaustion (≥10)** | 234 (65.2) | 123 (71.1) | 357 |
| **Low Emotional exhaustion (<10)** | 125 (34.8) | 50 (28.9) | 175 |
| **Total** | 359 | 173 | 532 |
| **Phi (φ)** |  |  | 0.059 |
| **p (Fisher’s exact test)** |  |  | **0.174** |
|  |  |  |  |
| **High Depersonalization (≥10)** | 154 (42.9) | 98 (56.6) | 252 |
| **Low Depersonalization (<10)** | 205 (57.1) | 75 (43.4) | 280 |
| **Total** | 359 | 173 | 532 |
| **Phi (φ)** |  |  | 0.129 |
| **p (Fisher’s exact test)** |  |  | **0.003*** |
|  |  |  |  |
| **Decreased personal accomplishment (≤10)** | 84 (23.4) | 37 (21.4) | 121 |
| **Mod-High personal accomplishment (>10)** | 275 (76.6) | 136 (78.6) | 411 |
| **Total** | 359 | 173 | 532 |
| **Phi (φ)** |  |  | -0.022 |
| **p (Fisher’s exact test)** |  |  | **0.659** |

*Supplementary Table 9. Living in conflict area*

|  | **No n (%)** | **Yes n (%)** | **Total** |
| --- | --- | --- | --- |
| **High Emotional exhaustion (≥10)** | 226 (63.5) | 131 (74.4) | 357 |
| **Low Emotional exhaustion (<10)** | 130 (36.5) | 45 (25.6) | 175 |
| **Total** | 356 | 176 | 532 |
| **Phi (φ)** |  |  | 0.11 |
| **p (Fisher’s exact test)** |  |  | **0.011*** |
|  |  |  |  |
| **High Depersonalization (≥10)** | 160 (44.9) | 92 (52.3) | 252 |
| **Low Depersonalization (<10)** | 196 (55.1) | 84 (47.7) | 280 |
| **Total** | 356 | 176 | 532 |
| **Phi (φ)** |  |  | 0.069 |
| **p (Fisher’s exact test)** |  |  | **0.111** |
|  |  |  |  |
| **Decreased personal accomplishment (≤10)** | 83 (23.3) | 38 (21.6) | 121 |
| **Mod-High personal accomplishment (>10)** | 273 (76.7) | 138 (78.4) | 411 |
| **Total** | 356 | 176 | 532 |
| **Phi (φ)** |  |  | -0.019 |
| **p (Fisher’s exact test)** |  |  | **0.742** |

*Supplementary Table 10. Verbal abuse*

|  | **No n (%)** | **Yes n (%)** | **Total** |
| --- | --- | --- | --- |
| **High Emotional exhaustion (≥10)** | 83 (36.4) | 212 (69.7) | 357 |
| **Low Emotional exhaustion (<10)** | 145 (63.6) | 92 (30.3) | 175 |
| **Total** | 228 | 304 | 532 |
| **Phi (φ)** |  |  | 0.065 |
| **p (Fisher’s exact test)** |  |  | **0.136** |
|  |  |  |  |
| **High Depersonalization (≥10)** | 96 (42.1) | 156 (51.3) | 252 |
| **Low Depersonalization (<10)** | 132 (57.9) | 148 (48.7) | 280 |
| **Total** | 228 | 304 | 532 |
| **Phi (φ)** |  |  | 0.091 |
| **p (Fisher’s exact test)** |  |  | **0.036*** |
|  |  |  |  |
| **Decreased personal accomplishment (≤10)** | 59 (25.9) | 62 (20.4) | 121 |
| **Mod-High personal accomplishment (>10)** | 169 (74.1) | 242 (79.6) | 411 |
| **Total** | 228 | 304 | 532 |
| **Phi (φ)** |  |  | -0.065 |
| **p (Fisher’s exact test)** |  |  | **0.144** |

*Supplementary Table 11. Physical abuse*

|  | **No n (%)** | **Yes n (%)** | **Total** |
| --- | --- | --- | --- |
| **High Emotional exhaustion (≥10)** | 296 (67.4) | 61 (65.6) | 357 |
| **Low Emotional exhaustion (<10)** | 143 (32.6) | 32 (34.4) | 175 |
| **Total** | 439 | 93 | 532 |
| **Phi (φ)** |  |  | -0.015 |
| **p (Fisher’s exact test)** |  |  | **0.732** |
|  |  |  |  |
| **High Depersonalization (≥10)** | 205 (46.7) | 47 (50.5) | 252 |
| **Low Depersonalization (<10)** | 234 (53.3) | 46 (49.5) | 280 |
| **Total** | 439 | 93 | 532 |
| **Phi (φ)** |  |  | 0.029 |
| **p (Fisher’s exact test)** |  |  | **0.568** |
|  |  |  |  |
| **Decreased personal accomplishment (≤10)** | 99 (22.6) | 22 (23.7) | 121 |
| **Mod-High personal accomplishment (>10)** | 340 (77.4) | 71 (76.3) | 411 |
| **Total** | 439 | 93 | 532 |
| **Phi (φ)** |  |  | 0.01 |
| **p (Fisher’s exact test)** |  |  | **0.787** |
